# Molecular identification and antifungal susceptibility profiles of clinical strains of *Fonsecaea* spp. isolated from patients with chromoblastomycosis in Rio de Janeiro, Brazil

**DOI:** 10.1371/journal.pntd.0006675

**Published:** 2018-07-26

**Authors:** Rowena Alves Coelho, Fábio Brito-Santos, Maria Helena Galdino Figueiredo-Carvalho, Juliana Vitoria dos Santos Silva, Maria Clara Gutierrez-Galhardo, Antonio Carlos Francesconi do Valle, Rosely Maria Zancopé-Oliveira, Luciana Trilles, Wieland Meyer, Dayvison Francis Saraiva Freitas, Rodrigo Almeida-Paes

**Affiliations:** 1 Laboratory of Mycology, Evandro Chagas National Institute of Infectious Diseases, Oswaldo Cruz Foundation (Fiocruz), Rio de Janeiro, RJ, Brazil; 2 Laboratory of Clinical Research in Infectious Dermatology, Evandro Chagas National Institute of Infectious Diseases, Oswaldo Cruz Foundation (Fiocruz), Rio de Janeiro, RJ, Brazil; 3 Molecular Mycology Research Laboratory, Centre for Infectious Diseases and Microbiology, Westmead Clinical School-Sydney Medical School, Marie Bashir Institute for Infectious Diseases and Biosecurity, University of Sydney, Westmead Institute for Medical Research, Sydney, Australia; University of Tennessee, UNITED STATES

## Abstract

**Background:**

Chromoblastomycosis (CBM) is a difficult-to-treat chronic subcutaneous mycosis. In Brazil, the main agent of this disease is *Fonsecaea pedrosoi*, which is phenotypically very similar to other *Fonsecaea* species, differing only genetically. The correct species identification is relevant since different species may differ in their epidemiologic aspects, clinical presentation, and treatment response.

**Methodology/Principal findings:**

Partial sequencing of the internal transcribed spacer (ITS) was used to identify twenty clinical isolates of *Fonsecaea* spp. Their *in vitro* antifungal susceptibility was determined using the broth microdilution method, according to the M38-A2 protocol. Amphotericin B (AMB), flucytosine (5FC), terbinafine (TRB), fluconazole (FLC), itraconazole (ITC), ketoconazole (KTC), posaconazole (POS), voriconazole (VRC), ravuconazole (RVC), caspofungin (CAS), and micafungin (MFG) were tested. The association between ITC/TRB, AMB/5FC, and ITC/CAS was studied by the checkerboard method to check synergism. The available patients’ data were correlated with the obtained laboratory results. *Fonsecaea monophora* (n = 10), *F*. *pedrosoi* (n = 5), and *F*. *nubica* (n = 5) were identified as CBM’ agents in the study. TRB and VRC were the drugs with the best *in vitro* activity with minimal inhibitory concentrations (MIC) lower than 0.25 mg/L. On the other hand, FLC, 5FC, AMB, and MFG showed high MICs. The AMB/5FC combination was synergistic for three *F*. *monophora* strains while the others were indifferent. Patients had moderate or severe CBM, and ITC therapy was not sufficient for complete cure in most of the cases, requiring adjuvant surgical approaches.

**Conclusions/Significance:**

*F*. *monophora*, the second most frequent *Fonsecaea* species in South America, predominated in patients raised and born in Rio de Janeiro, Brazil, without cerebral involvement in these cases. TRB, VRC, and the AMB/5FC combination should be further investigated as a treatment option for CBM.

## Author summary

Chromoblastomycosis is a disfiguring disease usually occurring in rural workers from poor and remote communities. In Brazil, the most frequent agents of this neglected disease are the species belonging to the genus *Fonsecaea*. The disease occurs after traumatic inoculation during work. As the lesions progress, itching becomes severe, and scratching may result in further inoculation to other body sites. When patients seek medical help, the lesions are usually extensive and disfiguring. For this reason, a more effective and less time-consuming treatment is important. Oral antifungal therapy is not very effective, must be taken for months or years, it is costly for most patients and often unavailable. Hence, it is important to determine the *in vitro* antifungal susceptibility and correlate it with the isolated species. In this study, *Fonsecaea monophora* was the predominant species and, differently from some studies, dissemination to the central nervous system was not observed. *In vitro* analysis showed that the most effective antifungal drugs were terbinafine and voriconazole, followed by itraconazole, the most used drug in the treatment of this disease. The combination of amphotericin B and flucytosine may be synergistic, depending on the infective strain.

## Introduction

Chromoblastomycosis (CBM) is a chronic fungal infection of cutaneous and subcutaneous tissues caused by traumatic implantation of several species of dematiaceous fungi [1]. In 2017, this mycosis was recognized as a neglected tropical disease by the World Health Organization [2].

The etiological agents of CBM belong mainly to the genera *Cladophialophora*, *Phialophora*, and *Fonsecaea* [3]. In the last decade, new species of the genus *Fonsecaea* have been described, based on molecular criteria: *Fonsecaea monophora* [4], *Fonsecaea nubica* [5], *Fonsecaea multimorphosa* [6], and *Fonsecaea pugnacius* [7]. These species can be found in nature, trace amounts in plant debris, thorns, and wood cortex, which provide microhabitats for these fungi [8]. In the Brazilian State of Maranhão, on the border of the Brazilian Amazon rainforest, several agricultural communities work on harvesting babassu (*Orbignya phalerata*), a wild palmacea specimen that was described as probable infection source in this area [9].

In the environment, all agents of CBM present in their mycelial form, which is composed by dematiaceous hyphae and conidia, which are specific for each genus. Infection usually follows a human trauma with a contaminated organic material such as plant thorns, wood, plant debris, grass, tree cortex among others, leading to the implantation of the fungus in the subcutaneous tissues, where the fungus changes to its parasitic form composed by muriform cells. These cells are heavily melanised and are extremely resistant to the harsh conditions imposed by the host immune system [10].

CBM can be caused by four *Fonsecaea* species: *F*. *pedrosoi*, *F*. *nubica* [4–6], *F*. *monophora* and *F*. *pugnacius*. The latter two show significant neurotropism, eventually leading to dissemination to the brain and other organs [4,7] or causing primary brain infection without skin lesions, which are classified as phaeohyphomycosis since no muriform cells are seen in tissues [10,11]. CBM may assume several clinical forms with different degrees of severity [10].

There is no treatment protocol to be followed, and antifungal therapy is often combined with physical methods such as cryosurgery or surgical excision for small lesions [12]. Itraconazole (ITC) and terbinafine (TRB) are the most used drugs in the treatment of CBM [10,13–15]. Other drugs used include posaconazole (POS), voriconazole (VRC), amphotericin B (AMB) and flucytosine (5FC) [10,16,17]. In addition, combined therapies of ITC with TRB [10,18], 5FC with AMB [17], or ITC with 5FC have been used [19].

It is important to determine the *in vitro* susceptibility of these isolates because of the difficulty found in the treatment of this mycosis and the frequency of refractory cases and relapses. The present study aimed to molecularly identify the species, to evaluate the *in vitro* susceptibility to antifungals, and to identify possible combinations of drugs with synergism against strains isolated from patients with CBM diagnosed in Rio de Janeiro state, Southeast Brazil, an area of low occurrence of this mycosis. Moreover, a clinical and laboratorial data association is provided for some patients.

## Material and methods

### Ethical statement

This study was approved by the Ethics Committee Board of the Evandro Chagas National Institute of Infectious Diseases (INI), Oswaldo Cruz Foundation (Fiocruz), under the number CAAE: 52247016.0.0000.5262.

### Fungal isolates

Twenty isolates of dematiaceous fungi from skin lesions of 17 patients with CBM were included in this study. These isolates were stored from 1999 to 2015 at the INI Mycology Laboratory and identified phenotypically as *Fonsecaea pedrosoi*. From the total of 17 patients, 12 were treated at the INI's Infectious Dermatology Outpatient Clinic, 7 of which were previously studied by Mouchalouat *et al*.,[20] and the remaining 5 were followed up at other institutions after mycological diagnosis at the INI (**Table 1**). All stored fungi were recovered on Sabouraud Dextrose Agar (Difco Laboratories, Sparks, MD, USA) incubated at 25°C for 10 days. Microscopically, hyphae were septate, branched, and brown staining with the predominance of conidiophores with short chains of smooth, thin-walled dematiaceous conidia.

**Table 1:** Demographics, clinical features, time of evolution and follow-up of CBM cases (20 strains from 17 patients).

N	Strain number(s)	Species	Sex^a^	Age	Occupation	State/region of Origin ^b^	City of Residence	Time of evolution	Severity	Outcome
**1**	16751–1	*F*. *pedrosoi*	M	No data available						
**2**	16451	*F*. *nubica*	M	No data available						
**3**^**c**^	19571/19889	*F*. *pedrosoi*	F	42	Maid	PB	Teresópolis	20 years	Moderate	Loss of Follow-up
**4**^**c**^	25543	*F*. *monophora*	M	72	Gardener	Portugal	Rio de Janeiro	32 years	Severe	Cure
**5**^**c**^	25811	*F*. *monophora*	M	65	Farmer	RJ	Bom Jardim	2 years	Severe	Transferred to another health unity
**6**^**c**^	28479	*F*. *nubica*	F	50	Housewife	ES	São João de Meriti	8 months	Moderate	Cure
**7**^**c**^	32999	*F*. *monophora*	F	36	Housewife	RJ	Rio de Janeiro	4 months	Moderate	Cure
**8**^**c**^	33420	*F*. *monophora*	M	45	Bricklayer	PB	Rio de Janeiro	9 months	Moderate	Cure
**9**	34113/34242	*F*. *nubica*	M	83	Gardener	MG	Itatiaia	10 years	Moderate	Loss of Follow-up
**10**	34904	*F*. *monophora*	M	No data available						
**11**	35962/36831	*F*. *monophora*	M	53	House Painter	PB	Rio de Janeiro	3 years	Severe	Cure
**12**	36134	*F*. *monophora*	M	35	Farmer	MG	Rio de Janeiro	No data available	Moderate	Cure
**13**	38437	*F*. *pedrosoi*	M	No data available						
**14**	38714	*F*. *pedrosoi*	M	No data available						
**15**	38833	*F*. *monophora*	M	60	Bricklayer	RJ	Rio de Janeiro	8 months	Moderate	Cure
**16**	41080	*F*. *monophora*	M	58	Bricklayer	PB	Rio de Janeiro	2 months	Moderate	Cure
**17**	48262	*F*. *nubica*	M	65	Snack bar attendant	CE	Rio de Janeiro	10 years	Severe	Cure

^a^ M: Male, F: Female.

^b^ State in Brazil where the patient was born, except patient 4, who was from Portugal. CE: Ceará, ES: Espírito Santo, MG: Minas Gerais, PB: Paraíba, RJ: Rio de Janeiro.

^c^ Cases 3–9 were previously reported by Mouchalouat *et al*. 2011.

### Molecular identification

Putative *Fonsecaea* spp. colonies were assessed on potato dextrose agar (PDA) (HiMedia Laboratories Pvt. Ltd., India) at 25°C after 7–14 days of inoculation. The DNA extraction and polymerase chain reaction (PCR) of the ITS1-5.8S-ITS2 region, the official Fungal DNA barcode, were performed according to Brito-Santos *et al*. [21]. PCR products were purified using the Wizard SV Gel and PCR Clean-Up System kit (Promega Corporation, Madison, USA) and sequenced at the Platform for DNA Sequencing PDTIS/Fiocruz. Sequences were edited using *Sequencher* 4.9 (Gene Codes Corporation, Ann Arbor, MI, USA), aligned and analyzed with MEGA 6.06 [22], and compared by BLAST with sequences available at the ISHAM ITS database (http://its.mycologylab.org). The molecular identification was considered valid when it presented more than 98.5% of identity, compared to the sequences available in the ISHAM-ITS database [23]. The evolutionary history was inferred by using the Maximum Likelihood method based on the Tamura-Nei model [24].

### Antifungal susceptibility testing

*In vitro* antifungal susceptibility testing was performed according to the recommendations proposed in the Clinical and Laboratory Standards Institute (CLSI) M38-A2 protocol [25] with modifications. AMB, FLC, ketoconazole (KTC), POS, ITC, VRC, ravuconazole (RVC), 5FC, TRB, caspofungin (CAS) (all from Sigma-Aldrich Chemical Corporation, St. Louis, MO, USA) and micafungin (MFG) (Astellas Pharma Tech Corporation, Takaoka city, Toyama, Japan) were tested. The inoculum was prepared from a seven-day old PDA culture; the cells were harvested in RPMI medium and diluted to approximately 0.4–5 × 10^4^ cells/mL. The plates were incubated at 35°C for five days [14]. The minimal inhibitory concentration (MIC) for AMB, FLC, KTC, POS, ITC, VRC, RVC, 5FC, and TRB; and the minimal effective concentration (MEC) for CAS and MFG were determined according to the CLSI M38-A2 protocol [25]. The reference strains *Aspergillus flavus* (ATCC 204304), *Aspergillus fumigatus* (ATCC 204305), *Candida krusei* (ATCC 6258) and *Candida parapsilosis* (ATCC 22019) were used for quality control.

### Antifungal combination

The susceptibility test with antifungal combinations was performed by the checkerboard method, where two different drugs were applied at different concentrations in a single 96-well plate, so that in each well there were different concentrations of the antifungals in combination. The concentrations assayed in the combinations were ITC 0.0075–4 mg/L with TRB 0.015–1 mg/L; AMB 0.0075–4 mg/L with 5FC 0.06–4 mg/L; CAS 0.0075–4 mg/L with ITC 0.06–4 mg/L. Drug interaction, classified according to the fractional inhibitory concentration index (FICI), which defines the type of interaction between the antifungal agents in combination, was as follows: synergism if FICI ≤ 0.5; indifference if 0.5 < FICI ≤ 4 and antagonism if FICI > 4 [26,27]. The FICI was obtained by the sum of the fractional inhibitory concentrations (FIC) or by the formula: FICI ═ (A/MIC (a)) + (B/MIC (b)), where: A = MIC of the drug (a) in combination; MIC (a) = MIC of drug (a) alone; B = MIC of the drug (b) in combination; MIC (b) = MIC of drug (b) alone [28].

### Statistical analyses

The geometric mean of MIC/MECs, MIC/MEC_50_, MIC/MEC_90_ and the MIC/MEC ranges were calculated using the Statistical Package for the Social Sciences v.17.0 (SPSS Inc, USA). Data analysis was performed in the GraphPad Prism 5 software. Kruskal-Wallis test was used to compare MIC of each antifungal drug between the different species. The Wilcoxon matched pairs test was used to compare MICs of two different drugs and the Friedman test to compare MICs of three or more antifungal drugs. *P* values lower than 0.05 were considered significant.

## Results

### Molecular identification

**Fig 1:**
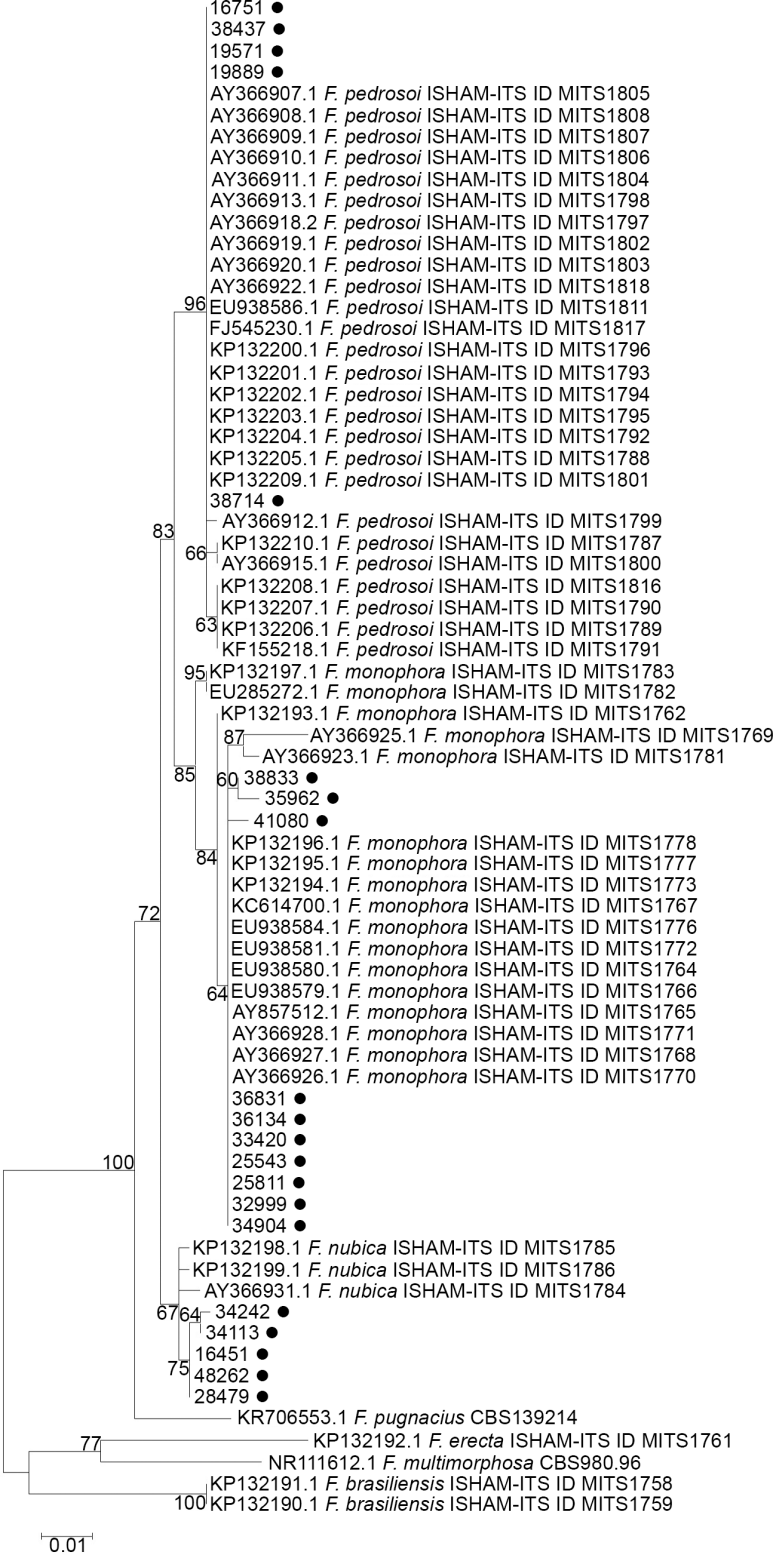
Molecular phylogenetic analysis using Maximum Likelihood. The percentage of trees in which the associated taxa clustered together is shown next to the branches. Initial tree(s) for the heuristic search were obtained automatically by applying Neighbor-Joining and BioNJ algorithms to a matrix of pairwise distances estimated using the Maximum Composite Likelihood (MCL) approach, and then selecting the topology with superior log likelihood value. The tree is drawn to scale, with branch lengths measured in the number of substitutions per site. The analysis involved 72 nucleotide sequences. All positions containing gaps and missing data were eliminated. Evolutionary analyses were conducted in MEGA 6 software [22]. Isolates of this study are marked with a filled circle. ITS Sequences of *Fonsecaea brasiliensis*, *Fonsecaea erecta*, and *Fonsecaea multimorphosa* were used as outgroups.

Ten isolates were identified as *F*. *monophora* (50%), five as *F*. *pedrosoi* (25%) and five as *F*. *nubica* (25%). The ITS sequencing alignment scores of the fungal isolates herein studied exhibited 99–100% identity compared with corresponding ITS sequences deposited in the ISHAM-ITS database **(Fig 1)**. The ITS sequences obtained during this study were deposited in NCBI/*GenBank* under the accession numbers MF616485 –MF616504.

### Antifungal susceptibility testing

Table 2 depicts the susceptibility profile of the strains included in this study. TRB (MIC range 0.015–0.25 mg/L) and VRC (MIC range 0.12–0.25 mg/L) were the antifungal drugs that showed the best *in vitro* activity against the *Fonsecaea* spp. isolates. FLC (MIC range 8–32 mg/L), 5FC (MIC range 2–32 mg/L), AMB (MIC range 4->16 mg/L) and echinocandins (MEC range 1–8 mg/L) showed higher MIC/MEC values. Overall, FLC was the azole with the poorest activity (*P*<0.0001) and among the echinocandins, CAS was more effective than MFG (*P* = 0.003). The susceptibility profile between the different species was very similar for the drugs tested. The few differences observed were as follows: *F*. *pedrosoi* presented MEC_90_ of 1 mg/L for MFG, while *F*. *monophora* and *F*. *nubica* presented both MEC_90_ of 8 mg/L (*P* = 0.0009) and *F*. *monophora* presented MIC_50_ and MIC_90_ of 8 mg/L for AMB, while *F*. *pedrosoi* and *F*. *nubica* presented MIC_50_ and MIC_90_ of 4mg/L for the same polyene drug (*P =* 0.0447). Although not significant (*P* = 0.0871), *F*. *nubica* presented MIC_90_ of 4 mg/L for 5FC, while for the other species the MIC_90_ was two-dilutions higher, that is, 16 mg/L.

**Table 2:** Minimal inhibitory concentrations (MIC) or minimal effective concentrations (MEC)^a^ of 11 antifungal drugs against 20 clinical isolates of *Fonsecaea* spp obtained from 17 different patients.

Antifungal drug	MIC/MEC^a^ (mg/L)															
	*Fonsecaea* spp. (n = 20)				*F*. *monophora* (n = 10)				*F*. *pedrosoi* (n = 5)				*F*. *nubica* (n = 5)			
	Range	MIC_50_^b^	MIC_90_	GM^c^	Range	MIC_50_	MIC_90_	GM	Range	MIC_50_	MIC_90_	GM	Range	MIC_50_	MIC_90_	GM
Amphotericin B	4->16	8	8	5.77	4->16	8	8	7.46	4–8	4	4	4.59	4->16	4	4	5.28
Ketoconazole	0.06–0.50	0.25	0.50	0.19	0.12–0.25	0.25	0.25	0.17	0.06–0.50	0.25	0.25	0.19	0.12–0.50	0.25	0.25	0.25
Fluconazole	8–32	16	16	12.55	8–16	16	16	11.31	8–16	16	16	12.13	8–32	16	16	16.00
Itraconazole	0.25–1	0.50	1	0.57	0.25–1	0.50	1	0.57	0.25–1	0.50	0.50	0.44	0.25–1	0.25	0.25	0.76
Posaconazole	0.06–0.50	0.12	0.50	0.16	0.06–0.25	0.12	0.25	0.13	0.06–0.50	0.25	0.25	0.19	0.12–0.50	0.25	0.25	0.21
Ravuconazole	0.25–1	0.50	1	0.64	0.25–1	0.50	1	0.62	0.25–1	0.50	0.50	0.50	0.50–1	1	1	0.87
Voriconazole	0.12–0.25	0.12	0.25	0.14	0.12–0.25	0.12	0.25	0.13	0.12–0.25	0.12	0.12	0.14	0.12–0.25	0.25	0.25	0.19
Flucytosine	2–32	8	16	6.28	2–16	8	16	6.50	4–32	16	16	10.56	2–4	4	4	3.48
Terbinafine	0.015–0.25	0.12	0.25	0.09	0.06–0.25	0.12	0.25	0.09	0.06–0.12	0.12	0.12	0.10	0.015–0.25	0.12	0.12	0.08
Caspofungin	1->8	2	4	1.73	1–4	2	4	1.74	2–2	2	2	2.00	1->8	2	2	2.00
Micafungin	1–8	8	8	5.66	8–8	8	8	8.00	1–8	1	1	2.00	8–8	8	8	8.00

^a^ The minimal effective concentrations (MEC) refer to caspofungin and micafungin. The minimal inhibitory concentrations (MIC) refer to the other antifungal drugs.

^b^ The MIC_50_ and MIC_90_ values correspond to the minimal inhibitory concentration of the antifungal able to inhibit the growth of 50 and 90% of all fungal isolates, respectively.

^c^ GM: Geometrical mean

### Antifungal combination

According to the FICI, when 5FC and AMB were tested in combination, synergistic interaction (FICI ≤ 0.5) was observed in 3 *F*. *monophora* isolates (30%). For the combinations ITC/TRB and ITC/CAS, an indifferent interaction (0.5 < FICI ≤ 4.0) was observed for all isolates tested. S1 Table depicts the results of the three combinations of antifungal drugs herein studied.

### Clinical and laboratorial correlation

It was possible to determine the probable site of infection for 9 out of the 12 patients with documented data, 8 of them in Rio de Janeiro and 1 in the Espírito Santo state. Among the patients infected in Rio de Janeiro, six were by *F*. *monophora* (75%) and two by *F*. *nubica* (25%). Regarding the geographic location, it is important to note that the three patients born and raised in Rio de Janeiro state were infected with *F*. *monophora* and all patients infected with *F*. *pedrosoi* were born outside the Rio de Janeiro state. In addition, two cases of *F*. *pedrosoi* without clinical data available (cases 13 and 14, **Table 1**) were diagnosed in patients from the Brazilian Amazon region.

Of the 12 patients followed up at INI, nine were cured; three of them used only antifungal drugs, two underwent surgical procedures, three used antifungal drugs associated with physical methods (cryosurgery and/or surgery) and one underwent surgery plus two sessions of cryosurgery (**Table 3**). The extension of treatment considering only the six patients who used antifungal drugs (ITC alone or in combination) ranged from 1 to 87 months (median = 9 months).

**Table 3:** Relationships between laboratorial and clinical data of the 12 patients followed up at INI/Fiocruz.

Case	Strain	Species	MIC^a^ (mg/L)			Treatment (months)				Total Time (months)
			ITC^b^	FLC^c^	TRB^d^	Initial	Change criterion	Subsequent	Outcome	
3	19571	*F*. *pedrosoi*	0.5	8	0.12	ITC 200 mg/day (8)	Slow improvement	ITC 400 mg/day (5)	Slow improvement	91
	19889		1	16	0.12			ITC 400 mg/day + FLC 200 mg/day (60) + cryosurgery (13 sessions)	Slow improvement	
								ITC 400 mg/day + TRB 250 mg/day (6)	Slow improvement	
								ITC 400 mg/day + TRB 500 mg/day (12)	Abandonment	
4	25543	*F*. *monophora*	0.5	16	0.12	ITC 400 mg/day + FLC 200 mg/day (18)	Improvement	ITC 200 mg/day + FLC 200 mg/day (4)	Cure	22
5	25811	*F*. *monophora*	0.25	8	0.06	ITC 400 mg/day (6)	Stroke not related to chromoblastomycosis	ITC 200 mg/day (1)	Transferred to other health unit	-
6	28479	*F*. *nubica*	0.25	16	0.12	Surgery	-	-	Cure	-
7	32999	*F*. *monophora*	0.25	8	0.06	Surgery	-	-	Cure	-
8	33420	*F*. *monophora*	1	8	0.06	ITC 200 mg/day (4.5)	Single lesion in immunosuppressed	Surgery	Cure	5
9	34113	*F*. *nubica*	1	32	0.12	ITC 200 mg/day (6)	Slow improvement	Cryosurgery (2 sessions)	Abandonment	10
	34242		1	16	0.25					
11	35962	*F*. *monophora*	0.5	16	0.25	Surgery	Lesion in immunosuppressed	Cryosurgery (2 sessions)	Cure	17
	36831		1	16	0.06					
12	36134	*F*. *monophora*	0.5	8	0.12	ITC 300 mg/day (2)	-	-	Cure	2
15	38833	*F*. *monophora*	1	16	0.12	ITC 200 mg/day (3)	Uncontrolled *diabetes mellitus*	Cryosurgery (18 sessions) + surgery	Cure	13
16	41080	*F*. *monophora*	0.5	16	0.12	ITC 300 mg/day (1)	-	-	Cure	1
17	48262	*F*. *nubica*	1	8	0.015	ITC 400 mg/day + TRB 250 mg/day (87)	Slow improvement	Cryosurgery + surgery	Cure	87

^a^ MIC: Minimal inhibitory concentration

^b^ ITC: itraconazole

^c^ FLC: fluconazole

^d^ TRB: terbinafine

Regarding the severity of the disease, of the nine patients infected by *F*. *monophora*, clinical information was available for eight, five of which were characterized by the moderate form and three with the severe form. Moderate and severe CBM was also observed in patients infected with *F*. *nubica*, and moderate CBM was observed in the patient infected by *F*. *pedrosoi* with available clinical data. The only case of cutaneous disseminated CBM was observed in a patient with *F*. *monophora*. The only case with tumor lesion was observed in another patient with *F*. *monophora*. Of the patients infected by *F*. *monophora*, seven were cured and one had no outcome information. Regarding the five patients with no clinical data, three were infected with *F*. *pedrosoi*, one with *F*. *monophora* and one with *F*. *nubica*.

Extracutaneous manifestations of the disease were not observed in any case, regardless of the isolated species.

## Discussion

This work represents a case series study of CBM for a period of 16 years in one of the main reference centers for infectious diseases in Rio de Janeiro. Studies on CBM in other Brazilian regions have been conducted in this decade by several groups [29–31], but in all these studies, data from Rio de Janeiro was missing. We believe that this study can be an important clinic-laboratorial contribution to the knowledge of the disease and an update to the actual Brazilian situation of CBM.

All four species of *Fonsecaea* up to now related to CBM are found in Brazil [5,7,32,33]. *F*. *pedrosoi* is the predominant species in South America, followed by *F*. *monophora* [34]. Rio de Janeiro, the geographic region where this study was conducted, is an area of low occurrence of CBM in Brazil [20], which explains the limited number of cases during the studied period. The high frequency of *F*. *monophora* in our study may indicate a reservoir for this species in this region. The fact that all patients born and raised in Rio de Janeiro were infected by *F*. *monophora* supports this hypothesis.

This is first study that evaluate *in vitro* antifungal susceptibility of CBM isolates in Rio de Janeiro. We found high MIC values for AMB corroborating other authors [35,36]. Treatment with AMB alone or combined with 5FC has not been used since the introduction of ITC during the 1980s. The frequent occurrence of nephrotoxicity, due to the drug characteristics and prolonged treatment [37,38], together with the reactivation of the infection with the drug discontinuation [10,34] are factors that hinder the use of AMB for CBM therapy.

The *Fonsecaea* strains included in this study presented low MIC values for KTC, similar to other studies [14,35,39]. This drug was the first systemic imidazole available, but it is rarely used due to serious hepatic reactions, as well as severe drug interactions [40,41]. Nowadays, ITC is considered the most commonly used drug for CBM treatment [10]. In this study, the MIC values for this drug indicate susceptibility of the isolates to the antifungal agent [42], and the schemes using ITC alone or associated with other antifungal or surgical modalities was able to lead 7 patients to cure.

POS, VRC and RVC represent the new generation of triazoles with a broad spectrum of activity and a favourable pharmacokinetic profile [43]. POS is known to have a better *in vitro* activity than ITC against clinical *Fonsecaea* isolates [44] in accordance with the results of this study. VRC has good *in vitro* activity against CBM agents, including *Fonsecaea* spp. [45]. However, in addition to its high cost [46], the prescription of VRC should be done with caution, since it presents risk of photo toxicity and cutaneous carcinoma in prolonged periods of treatment [47]. To the best of our knowledge, this is the first study on *Fonsecaea* spp. susceptibility to RVC using the CLSI M38-A2 protocol. González *et al*. [48] reported the *in vitro* activity of RVC against isolates of *F*. *pedrosoi* with MICs ranging between 0.125–0.5 mg/L using M38-A CLSI protocol. Despite the use of another protocol, this work reports MIC values ≤ 1 mg/L for the same drug against clinical *Fonsecaea* isolates. However, based on our results that showed POS and VRC with a better *in vitro* profile than RVC, the first two azoles should be considered in the treatment of CBM, instead of RVC.

The MIC values found for 5FC and FLC were compatible with other studies, showing that these drugs are ineffective *in vitro* against *Fonsecaea* spp., discouraging their use in CBM treatment [35,39,44]. As for 5FC, its use in the mid-1960s marked the beginning of chemotherapy approaches for CBM [45]. However, it was later observed that *F*. *pedrosoi* is able to develop *in vitro* resistance to 5FC [45,49–53]. Although not prohibited, the drug is not registered in the Brazilian regulatory agency (ANVISA) and is not commercialized on the Brazilian market [54], because there is no pharmaceutical industry that manufactures this antifungal drug in our country, which leads to a need to its import by tertiary hospitals.

Several studies have reported the susceptibility of *Fonsecaea* spp. to TRB, revealing its potent action against various filamentous fungi and in the treatment of CBM, demonstrating up to 80% of cure rate [14,39,55,56]. Our results were consistent with those studies showing low MIC values for this drug. In addition, TRB shows a potent antifibrotic effect in recent lesions [57]. This drug has little affinity for the cytochrome P450 enzyme system, resulting in less interaction with other substances [58] and in a general way, is well-tolerated, indicating an effective option for the treatment of CBM.

Echinocandins have a limited role in the treatment of CBM due to high MEC values for *F*. *monophora* and *F*. *nubica*, as observed in other studies [44,59]. Isolates of *F*. *pedrosoi* presented a better *in vitro* response to MFG. Nevertheless, due to the low number of analysed isolates, it is suggested that further studies will assess whether this echinocandin is specifically effective against *F*. *pedrosoi*.

According to some studies, in particular CBM cases, the best therapeutic strategy would be the association of two antifungals based on the results of previous susceptibility tests [17]. Some of the suggested combinations are AMB and 5FC [45,60] or ITC and TRB [61]. Our work showed a synergism between AMB and 5FC in three *F*. *monophora* isolates. This combination has two distinct mechanisms enhancing the antifungal action: AMB binds to ergosterol of the fungus membrane forming pores and 5FC acts inhibiting the synthesis of nucleic acids. This combination is widely used in cases of cryptococcal meningitis because it has a more effective penetration into the SNC [62]. In the past, the combination AMB/5FC had been used for CBM treatment [17,63,64], but now a days it is no longer considered due to the adverse side effects [10]. However, we believe that this association could be beneficial in severe cases of CBM, especially those with brain involvement. The synergism found for 100% of the isolates of *Phialophora verrucosa* by Li *et al*. [18] encouraged the use of ITC and CAS combination in our study. A single study [65] found synergism for isolates of *F*. *monophora*. An indifferent interaction was observed in all *Fonsecaea* isolates of this study, which is compatible with most studies [15,17,61].

There are few studies comparing *in vitro* susceptibility among clinical *Fonsecaea* isolates. Najafzadeh *et al*. [44] found no significant differences among species in the activity of eight antifungals (AMB, FLC, ITC, VRC, POS, CAS, anidulafungin and isavuconazole) against *F*. *pedrosoi*, *F*. *monophora* and *F*. *nubica*. However, in this study, *F*. *monophora* showed higher MIC values than *F*. *nubica* for AMB (*P* = 0.0447), and the species *F*. *monophora* and *F*. *nubica* (*P* = 0.0009) had higher MEC values for MFG when compared to *F*. *pedrosoi*.

CBM is known as a difficult-to-treat disease, there is no standard drug of choice and relapses are frequent [10,34,45,57,66]. There are many factors that can influence the patient outcome that can be related to the host or the fungal species. The host immune response, local lymphedema, and fibrosis become a barrier for a proper drug bioavailability at the site of infection. In addition, muriform cells are heavily pigmented and represent a resistant fungal form against antimicrobial compounds [67]. In the same vein, there is no clear correlation between *in vitro* susceptibility and clinical practice [45]. In general, MIC values ≤ 1 mg/L usually indicate a potential susceptibility of most drugs used in the treatment of infections by dematiaceous fungi [14,42], as occurred in this study. It is also possible that the broth microdilution test is not the best method to guide therapeutic management in CBM. In fact, in a correlation study between different antifungal susceptibility methodologies and clinical outcome of cryptococcosis patients treated with AMB showed that time-kill assays are more suitable to predict treatment failure than broth microdilution and gradient diffusion methods [68]. Further studies are necessary to check if a similar scenario occurs in CBM.

Due to the hardships to obtain the parasitic form of the CBM agents *in vitro* [67,69], most authors perform antifungal susceptibility testing using conidia. This is not an invalid strategy, since a similar scenario occurs in sporotrichosis, another deep mycosis, for what the official antifungal susceptibility testing guidelines suggest the use of conidia instead of the parasitic yeast-like form [25]. However, we are aware that this can be a bias in the correlation of *in vitro* and *in vivo* results.

We were not able to observe clear relationships between the treatment responses and the antifungal susceptibility of the isolates. ITC was used in almost all clinical cases, because it is distributed free of charge in our institution. However, most of patients only presented a slight improvement with this drug, despite the MIC observed. It is common the development of fibrosis in lesions of CBM [70], what hinders the action of the drugs, since it prevents their penetration [71]. In addition, ITC needs an acidic gastric environment to be properly absorbed [72]. A decrease in the production of gastric juice may result in a higher pH of the stomach, thus reducing the bioavailability of this drug and therefore its activity [73,74]. No synergism was observed between ITC and TRB in this study and the two cases treated with this drug combination required surgical approaches for complete cure. In a study with CBM cases, to which drugs were administered together for a long period of time, failure was observed. So, the authors chose to weekly alternate these drugs, with a positive outcome in some cases [61].

In summary, this study demonstrated that TRB and VRC exhibited better *in vitro* activity against *Fonsecaea* spp., while AMB, FLC, 5FC and echinocandins played a limited role in the CBM treatment because of their relatively high MICs. However, AMB and 5FC presented *in vitro* synergism for a few strains, which may be useful as a salvage therapy. ITC, although with higher MICs, were used alone or in association and lead to cure in moderate to severe clinical cases. Despite the fact that we did not use TRB as the sole therapeutic drug in the patients herein described, we believe that more attention should be given to this antifungal in the context of CBM treatment, due to the low MIC values observed in this study as well as safety and effectiveness in other studies [56].

Our work provides perspectives for future studies of clinical follow-up, treatment and outcome of patients with CBM, as well as the determination of *in vitro* susceptibility to antifungal and new compounds with fungicidal action, especially in melanized fungi.

## Supporting information

S1 Table Fractional inhibitory concentration index values of three antifungal combinations for clinical isolates of *Fonsecaea* spp. (DOCX)
