# Supplementary material for: Molecular identification and antifungal susceptibility profiles of clinical strains of Fonsecaea spp. isolated from patients with chromoblastomycosis in Rio de Janeiro, Brazil
Source: PLoS Negl Trop Dis. 2018 Jul 26;12(7):e0006675. doi: 10.1371/journal.pntd.0006675 (PMC6080783; doi:10.1371/journal.pntd.0006675)
Supplement: S1 Table — (DOCX) [file pntd.0006675.s001.docx]

S1 Table: Fractional Inhibitory Concentration Index values of three antifungal combinations for clinical isolates of *Fonsecaea* spp.

| Strains | MIC (µg/mL) of the following drugs | | | FICI TRB/ITZ | MIC (µg/mL) of the following drugs | | | FICI 5FC/AMB | MIC (µg/mL) of the following drugs | | | FICI ITZ/CAS |
| --- | --- | --- | --- | --- | --- | --- | --- | --- | --- | --- | --- | --- |
|  | TRB | ITZ | TRB/ITZ |  | 5FC | AMB | 5FC/AMB |  | ITZ | CAS | ITZ/CAS |  |
| 16751 | 0.03 | 0.25 | 0.015/0.5 | 2.50 | 4 | 4 | 4/2 | 1.50 | 0.25 | 2 | 0.25/2 | 2 |
| 16451 | 0.25 | 1 | 0.12/1 | 1.50 | 4 | 4 | 4/2 | 1.50 | 1 | ≥16 | 0.5/1 | 0.5625 |
| 19571 | 0.12 | 0.5 | 0.03/0.5 | 1.25 | 16 | 4 | 4/2 | 0.75 | 0.5 | 2 | 0.25/2 | 1.50 |
| 19889 | 0.12 | 1 | 0.06/0.5 | 1 | 16 | 4 | 1/4 | 1.0625 | 1 | 4 | 0.25/2 | 0.75 |
| 25543 | 0.12 | 0.5 | 0.06/0.12 | 0.75 | 2 | 4 | 1/2 | 1 | 0.5 | 2 | 0.25/2 | 1.50 |
| 25811 | 0.12 | 0.25 | 0.06/0.25 | 1.50 | 2 | 8 | 4/2 | 2.25 | 0.25 | 4 | 0.25/2 | 1.50 |
| 28479 | 0.12 | 0.25 | 0.06/0.25 | 1.50 | 4 | ≥16 | 2/4 | 0.75 | 0.25 | 2 | 0.25/2 | 2 |
| 32999 | 0.12 | 0.25 | 0.06/0.25 | 1.50 | 8 | 8 | 0.25/4 | 0.53125 | 0.25 | 2 | 0.25/2 | 2 |
| 33420 | 0.12 | 1 | 0.06/0.5 | 1 | 8 | 8 | 0.06/4 | 0.5078125 | 1 | 2 | 0.25/2 | 1.25 |
| 34113 | 0.12 | 1 | 0.06/0.5 | 1 | 4 | 4 | 2/2 | 1 | 1 | 2 | 0.5/0.5 | 0.75 |
| 34242 | 0.12 | 1 | 0.12/0.5 | 1.50 | 4 | 8 | 4/2 | 1.25 | 1 | 4 | 0.5/2 | 1 |
| 34904 | 0.12 | 1 | 0.12/0.25 | 1.25 | 8 | ≥16 | 1/4 | 0.375 | 1 | 4 | 0.5/1 | 0.75 |
| 35962 | 0.12 | 0.5 | 0.03/0.5 | 1.25 | 16 | 8 | 1/4 | 0.5625 | 0.5 | 4 | 0.25/2 | 1 |
| 36134 | 0.25 | 1 | 0.03/0.5 | 0.625 | 8 | ≥16 | 1/4 | 0.375 | 1 | 4 | 0.25/2 | 0.75 |
| 36831 | 0.12 | 0.5 | 0.06/0.25 | 1 | 16 | 8 | 2/2 | 0.375 | 0.5 | 4 | 0.25/2 | 1 |
| 38437 | 0.12 | 0.25 | 0.06/0.25 | 1.50 | 32 | 8 | 4/4 | 0.625 | 0.25 | 2 | 0.25/2 | 2 |
| 38714 | 0.12 | 0.5 | 0.06/0.5 | 1.50 | 4 | 4 | 2/2 | 1 | 0.5 | 2 | 0.25/2 | 1.50 |
| 38833 | 0.12 | 1 | 0.06/0.12 | 0.625 | 8 | 8 | 4/4 | 1 | 1 | 2 | 0.25/1 | 0.75 |
| 41080 | 0.12 | 0.5 | 0.06/0.12 | 0.75 | 4 | 8 | 4/2 | 1.25 | 0.5 | 2 | 0.25/2 | 1.50 |
| 48262 | 0.03 | 1 | 0.015/0.5 | 1 | 2 | 4 | 0.06/4 | 1.03125 | 1 | 2 | 0.25/2 | 1.25 |

TRB: terbinafine; ITZ: itraconazole; TRB/ITZ: terbinafine in combination with itraconazole; 5-FC: flucytosine; AMB: amphotericin B; 5-FC/AMB: flucytosine in combination with amphotericin B; CAS: caspofungin; ITZ/CAS: itraconazole in combination with caspofungin.*16 mg/L for those with MIC >8 mg/L was considered in the FICI calculation.
